# Expression of genes involved in neurogenesis, and neuronal precursor cell proliferation and development: Novel pathways of human ovarian granulosa cell differentiation and transdifferentiation capability *in vitro*

**DOI:** 10.3892/mmr.2020.10972

**Published:** 2020-01-31

**Authors:** Maciej Brązert, Wiesława Kranc, Piotr Celichowski, Maurycy Jankowski, Hanna Piotrowska-Kempisty, Leszek Pawelczyk, Małgorzata Bruska, Maciej Zabel, Michał Nowicki, Bartosz Kempisty

**Affiliations:** 1 Division of Infertility and Reproductive Endocrinology, Department of Gynecology, Obstetrics and Gynecological Oncology, Poznań University of Medical Sciences, 60-535 Poznań, Poland; 2 Department of Anatomy, Poznań University of Medical Sciences, 60-781 Poznań, Poland; 3 Department of Histology and Embryology, Poznań University of Medical Sciences, 60-781 Poznań, Poland; 4 Department of Toxicology, Poznań University of Medical Sciences, 60-631 Poznań, Poland; 5 Division of Histology and Embryology, Department of Human Morphology and Embryology, Wrocław Medical University, 50-368 Wrocław, Poland; 6 Division of Anatomy and Histology, University of Zielona Góra, 65-046 Zielona Góra, Poland; 7 Department of Obstetrics and Gynecology, University Hospital and Masaryk University, 625 00 Brno, Czech Republic

**Keywords:** human granulosa cells, *in vitro* culture, differentiation, neural precursor cell proliferation

## Abstract

The process of neural tissue formation is associated primarily with the course of neurogenesis during embryonic life. The source of neural-like cells is stem cells, which, under the influence of appropriate differentiating factors, may differentiate/transdifferentiate towards a neural-like lineage. The present study suggested that, under long-term *in vitro* culture conditions, human ovarian granulosa cells (GCs), obtained from granulosa-rich follicular fluid, acquired new properties and expressed genes characteristic of the ontological groups ‘neurogenesis’ (GO:0022008), ‘neuronal precursor cell proliferation’ (GO:0061351) and ‘nervous system development’ (GO:0007399), which are closely related to the formation of neurons. The present study collected GCs from 20 women referred for the procedure of *in vitro* fertilization. Cells were maintained in long-term *in vitro* culture for 30 days, and RNA was isolated after 1, 7, 15 and 30 days of culture. The expression profile of individual genes was determined using the Affymetrix microarray method. The 131 genes with the highest expression change in relation to day 1 of culture were then selected; the 10 most affected genes found to be primarily involved in nerve cell formation processes were chosen for consideration in this study: *CLDN11, OXTR, DFNA5, ATP8B1, ITGA3, CD9, FRY, NANOS1, CRIM1* and *NTN4*. The results of the present study revealed that these genes may be considered potential markers of the uninduced differentiation potential of GCs. In addition, it was suggested that GCs may be used to develop a cell line showing neuronal characteristics after 30 days of cultivation. In addition, due to their potential, these cells could possibly be used in the treatment of neurodegenerative diseases, not only in the form of ‘cultured neurons’ but also as producers of factors involved in the regeneration of the nervous system.

## Introduction

The main function of ovarian granulosa cells (GCs) under physiological conditions is maintenance of the proper courses of folliculogenesis and oogenesis (1). In recent years, it has been demonstrated that human (h)GCs exhibit stem-like properties under conditions of long-term *in vitro* culture; therefore, they can differentiate into other cell types, such as osteoblasts, chondrocytes and muscle cells, under the influence of appropriate factors (2–4). A number of previous studies seem to suggest that GCs may also differentiate towards neural cells, despite the lack of any obvious link between these two cell types (5).

For a number of years, it was thought that the process of neurogenesis occurs only during embryonic and perinatal stages in mammals (6). Neurogenesis is defined as the process of functional neuron generation from precursor cells (7). During embryogenesis, the neural plate is formed, with its invaginations built from neuroepithelium. In subsequent stages of development, the neuroepithelium lines the inner layer of the neural tube, which gives rise to specific parts of the central nervous system. A characteristic feature of neuroepithelial cells is the ability to proliferate rapidly, resulting in separation of the sub-ventricular zone, which is the center of the brain ventriculi. The sub-ventricular zone contains numerous multipotent stem cells that are capable of transforming into neuron, astrocyte or oligodendrocyte progenitors (8). In the subsequent stages of brain development, neuroblasts are transformed into proneurons that move along radial astrocytes to the developing brain and spinal cord (9). Neurons of the peripheral nervous system arise from multipotent neural crest cells that migrate to target sites in an intercellular manner. At the destination, one neuronal tip becomes the next axon with the growth cone at the end. This cone has the ability to move in a quadriplegic motion towards the innervated organ. The complex process of neurogenesis depends on a number of factors, including the key role played by proteins belonging to the transforming growth factor β (TGFβ) family (10,11).

One of the main goals of numerous research groups is the production of stable, functional neural cell lines that may be used to restore damage to the nervous system. In recent years, a number of attempts have been made to differentiate cells of stem-like potential towards neural lineage (5). These studies primarily used induced pluripotent stem cells (iPSCs) (12–14). Despite the difficulties, an increasing number of scientists are trying to find more sources of functional nerve cells (15–18). The search has moved away from the model based on cells taken from the embryo, as well as iPSCs, as this is considered an unstable model that exhibits a tendency towards tumorigenesis (19). New research focuses primarily on the reprogramming of stem-like somatic cells towards neurons.

There are a number of reports on the possibility of differentiation of mesenchymal stem cells towards neural stem cells (5,20,21). Herman *et al* (20) was the first to present six protocols describing the reprogramming of bone marrow-derived human mesenchymal stem cells into neural stem cells. The process of this differentiation was possible by adding appropriate growth factors including brain-derived neurotrophic factor, platelet-derived growth factor, epidermal growth factor, fibroblast growth factor 2 and retinoic acid to the culture medium (14,20). Another important model for obtaining neuronal cells is the transdifferentiation of somatic cells. Transdifferentiation is the reprogramming of somatic cells into another cell type, omitting the pluripotent stem cell stage. Transdifferentiation of somatic cells towards neurons seems to be particularly important. This method could become a novel strategy in acquiring neural cells, creating new treatment options for neurodegenerative diseases, and injuries to the central or peripheral nervous system (22). Recent studies have indicated that stem cells could become a tool for the effective treatment of neurological conditions (23–25). The use of stem cells can have a number of positive effects not necessarily associated with the artificial growth of new nerves. Mesenchymal stem cells in particular could be used to treat a number of neurodegenerative diseases, including Parkinson's disease, Alzheimer's disease and age-related macular degeneration, as well as traumatic brain injury and glioblastoma (24,26). Parkinson's disease manifests as a movement disorder due to loss of the substantia nigra dopaminergic neurons. In recent years, one of the most promising tools for effective treatment of this disease is based on the application of stem cells within the striatum (27). Stem cell therapy has also been used to treat stroke. Administration of stem cells within the first 24 h significantly increases the chances of patient recovery (28). In addition, cell therapy has an immunosuppressive and angiogenic effect; therefore, patients have improved health after the application of stem cells.

Another important issue, the mechanisms of which are not yet fully elucidated, is neurogenesis in adult mammals. For a long time, studies have indicated that new neurons are formed in the subgranular zone of the dentate gyrus of the mammalian hippocampus (29,30) during learning, remembering or stress (31). Neurogenesis in adults is often discussed and it is believed that ~700 new neurons are created every day in the cusp of the human hippocampus (32,33), while other studies have suggested that the neurogenesis process decreases with age (34,35). It is suggested that the number of developing progenitor cells in the dentate gyrus drops sharply in the first year of life, as in healthy adults and patients with neurological pathology (epilepsy) no ‘young’ neurons have been detected in the hippocampal dentate gyrus (30). A previous rodent study indicated that mice maintained in a sensory enriched environment exhibited significantly more new neurons in the hippocampus than mice bred in cages (36).

The main objective of the present study was to identify the potential molecular markers characteristic of GC differentiation, and to develop a cell line possessing neuronal characteristics after 30 days of cultivation. The new properties of hGCs may also be used in the context of reconstruction of tissues following injury. In the present study, the most important finding was the identification of genes that perform a large role in the process of nerve cell formation during long-term *in vitro* culture. This knowledge might serve as a basic molecular entry into further *in vitro* and clinical studies.

## Materials and methods

### 

#### Previous work

Part of the materials and methods is based on other publications from the same research team, presenting results from the cycle of studies related to human ovarian GCs (37–41).

#### Patient clinical evaluations and GC collection

A total of 20 female patients (18–40 years; mean age, 27 years) assigned to the procedure of *in vitro* fertilization (IVF) at the Division of Infertility and Reproductive Endocrinology, Poznań University of Medical Sciences (Poznań, Poland) qualified for the present study. Patients were recruited between May 2017 and August 2019. Patients underwent controlled ovarian hyperstimulation with human recombinant follicle-stimulating hormone (rFSH; Gonal-F^®^; Merck KGaA) and highly purified human menopausal gonadotropin (hMG-HP; Menopur^®^; Ferring B.V.) and gonadotropin-releasing hormone (GnRH) antagonist (Cetrotide^®^; Merck KGaA). Ovulation was induced by subcutaneous injection of 6,500 IU human chorionic gonadotropin (hCG; Ovitrelle^®^; Merck KGaA). The doses of gonadotropins and GnRH antagonist were controlled and recorded for every patient. The follicular fluid (FF) containing oocytes and GCs was collected and transferred to a qualified embryologist who extracted all oocytes from the fluid and passed it on to the further stages of IVF. At this point, it was re-verified that FF did not contain oocytes. Subsequently, the GC-containing FF (this sample is usually discarded at this stage) was transferred for further laboratory testing. The FF containing GCs was collected during transvaginal ultrasound-guided oocyte pickup, 36 h after administration of hCG. GCs suspended in FF were obtained from follicles with a diameter >16 mm.

The exclusion criteria for the study were: i) A potential risk of inadequate ovarian stimulation according to the Bologna criteria of poor ovarian responders, published by the European Society of Human Reproduction and Embryology in 2011 (42); ii) serum antimullerian hormone 0.7 ng/ml as a cut-off value; iii) patients with a serum level of FSH >15 mU/ml on the 2nd-3rd day of the cycle; iv) patients with polycystic ovary syndrome; or v) patients with endometriosis. The present study was approved by resolution 558/17 of the Poznań University of Medical Sciences Bioethical Committee. All participating patients were informed about the course of the study and expressed their written consent to use the material collected from them during the IVF procedure.

#### Long-term primary in vitro cell culture

The GC-containing FF was washed twice using Dulbecco's phosphate-buffered saline (Sigma-Aldrich; Merck KGaA) and centrifuged at 200 × g for 10 min at room temperature. The culture medium consisted of Dulbecco's modified Eagle's medium (Sigma-Aldrich; Merck GaA), 2% fetal bovine serum (Sigma-Aldrich; Merck KGaA), 4 mM L-glutamine (stock 200 mM; Gibco; Thermo Fisher Scientific, Inc.), 10 mg/ml gentamicin (Gibco; Thermo Fisher Scientific, Inc.), 10,000 U/ml penicillin and 10,000 µg/ml streptomycin (Gibco; Thermo Fisher Scientific, Inc.). GCs were cultivated at 37°C under aerobic conditions (5% CO_2_). The cultures used samples in which necrotic and apoptotic cells accounted for <5%. Once adherent cells were >90% confluent, they were detached with 0.05% trypsin-EDTA (Gibco; Thermo Fisher Scientific, Inc.) for 1–3 min and counted using a fluorescence automatic cell counter (ADAM-MC; NanoEnTek America, Inc.). GCs were then cultivated for 30 days; total RNA was isolated after 1, 7, 15 and 30 days (38,39,43). The medium was replaced every 72 h. Cell morphology was observed after 1, 7, 15 and 30 days of culture under an inverted light microscope (Olympus IXC73; Olympus Corporation).

#### Total RNA isolation

Total RNA was isolated after 1, 7, 15 and 30 days of culture. The Chomczyński-Sacchi method was used for total RNA extraction (44). The GCs, after trypsin treatment, were suspended in a 1-ml mixture of guanidine thiocyanate and phenol in monophase solution (TRI Reagent^®^; Sigma-Aldrich; Merck KGaA); 1 ml TRI Reagent^®^ was used to lyse 5–10×10^6^ cells, with subsequent storage of the samples at −80°C. After thawing, 0.2 ml chloroform per ml TRI Reagent^®^ was added to the samples, which were then gently mixed (15 sec), and left to stand for 15 min at room temperature. After centrifugation (12,000 × g, 15 min, room temperature), three phases were visible: Red organic phase (containing protein), interphase (containing DNA) and colorless upper phase (containing RNA). The aqueous phase that contained the RNA was precipitated with 0.5 ml 2-propanol (cat. no. I9516; Sigma-Aldrich; Merck KGaA) per ml TRI Reagent^®^ and incubated for 10 min at room temperature. Finally, the RNA pellet was washed with 75% ethanol. The resulting RNA was used for further analysis. The total mRNA was determined from the optical density at 260 nm, and the RNA purity was estimated using the 260/280 nm absorption ratio (NanoDrop spectrophotometer; NanoDrop; Thermo Fisher Scientific, Inc.). Only samples with a 260/280 absorbance ratio >1.8 were used in the present study.

#### Microarray expression analysis

Total RNA (100 ng) from each pooled sample was subjected to two rounds of sense cDNA amplification (Ambion WT Expression kit; Thermo Fisher Scientific, Inc.), according to the manufacturer's protocol. The obtained cDNA was used for biotin labeling and fragmentation using the Affymetrix GeneChip WT Terminal Labeling and Hybridization kit (Affymetrix; Thermo Fisher Scientific, Inc.). Biotin-labeled fragments of cDNA (5.5 µg) were hybridized to the Affymetrix Human Genome U219 Array (HgU 219; 48°C/20 h; Affymetrix; Thermo Fisher Scientific, Inc.). Microarrays were then washed and stained according to the technical protocol using the Affymetrix GeneAtlas Fluidics Station (Affymetrix; Thermo Fisher Scientific, Inc.). The array strips were scanned using the Imaging Station of the GeneAtlas system (Affymetrix; Thermo Fisher Scientific, Inc.). Preliminary analysis of the scanned chips was performed using Affymetrix GeneAtlas Operating Software v. 2.0.0.460 (Affymetrix; Thermo Fisher Scientific, Inc.). The quality of gene expression data was confirmed according to the quality control criteria provided by the software. The obtained CEL files were imported into downstream data analysis software (38,45).

#### Reverse transcription-quantitative (RT-q)PCR

RT-qPCR was performed to validate microarray results, using the same cDNA samples. A total of 20 genes were selected from the 131 selected genes: 10 represent the highest change in expression, and 10 represent the lowest change in expression, but all 20 had been upregulated in relation to day 1 of primary culture. Each reaction was repeated three times, with three replicates per group. For RT, 1 µg each RNA sample was used. RT was conducted based on the protocols and reagents of the RT^2^ First Stand kit (cat. no. 330401; Qiagen, Inc.), using a Veriti 96-well Thermal Cycler (Applied Biosystems; Thermo Fisher Scientific, Inc.). PCR was performed using the Light Cycler^®^ 96 (Roche Diagnostics GmbH), RT^2^ SYBR Green ROX qPCR Master Mix (Qiagen, Inc.) and sequence-specific primers (Table I). The reaction cocktail used for the test contained 3 µl nuclease-free water (Invitrogen; Thermo Fisher Scientific, Inc.), 5 µl RT^2^ SYBR Green ROX qPCR Master Mix (Qiagen, Inc.), 0.5 µl forward primers, 0.5 µl reverse primers and 1 µl cDNA. *GAPDH*, β-actin (*ACTB*) and hypoxanthine phosphoribosyltransferase 1 (*HPRT1*) were used as reference genes. Thermocycling conditions were as follows: Preincubation at 37°C for 30 sec; 3-step amplification (95°C for 15 sec, 58°C for 15 sec, 72°C for 15 sec) for 45 cycles; melting (95°C for 60 sec, 40°C for 60 sec, 70°C for 1 sec, 95°C for 1 sec); cooling at 37°C for 30 sec. Gene expression was analyzed using the 2^−ΔΔCq^ method. The qPCR primers were designed using Primer3Plus software (http://primer3plus.com/cgi-bin/dev/primer3plus.cgi).

#### Statistical analysis

All of the presented analyses and graphs of microarray expression were performed and generated using Bioconductor (v. 3.10; http://www.bioconductor.org/) and R programming language (v 3.5.1; www.r-project.org). Each CEL file was merged with a description file. In order to correct background, normalize and summarize results, the Robust Multiarray Averaging algorithm was used. To determine the statistical significance of the analyzed genes, moderated t-statistics from the empirical Bayes method were performed. The obtained P-value was corrected for multiple comparisons using Benjamini and Hochberg's false discovery rate. The selection of significantly altered genes was based on P<0.05 and expression >2-fold. The differentially expressed gene list (separated for up- and downregulated genes) was uploaded to the Database for Annotation, Visualization and Integrated Discovery (DAVID, v 6.8) software to investigate their mutual relations (46–48). DAVID was used for extraction of the genes belonging to ‘neurogenesis’, ‘neuronal precursor cell proliferation’ and ‘nervous system development’ GO BP terms. Up- and downregulated gene sets were subjected to the DAVID search separately and only gene sets with adjusted P<0.05 were selected.

Subsequently, mutual interactions between the genes belonging to the selected Gene Ontology (GO) biological process (BP) terms were investigated using the GOplot package (49). Finally, the functional interactions (FIs) between genes that belong to the chosen GO BP terms were investigated by REACTOME FIViz application in the Cytoscape 3.6.0 software (https://cytoscape.org/). The ReactomeFIViz application is designed to find pathways and network patterns related to cancer and other types of diseases. This application accesses the pathways stored in the Reactome database, allowing pathway enrichment analysis for a set of genes, visualizing hit pathways using manually laid-out pathway diagrams directly in Cytoscape, and investigating functional relationships among genes in hit pathways. The application can also access the Reactome FI network, a highly reliable, manually curated pathway-based protein FI network covering >60% of human proteins. The results of the experiments refer to three separate biological replicates, and represent the average measurements (mean ± standard error of mean) from each time period of the cell cultures and mRNA measurements, as determined by RT-qPCR. In the case of RT-qPCR, biological replicates were divided into three technical repetitions. As an internal control, *HPRT1, GAPDH* and *ACTB* were used, for which the levels of transcripts analyzed were standardized in each sample. Relative quantification was performed using the 2^−ΔΔCq^ method to determine target cDNA quantification (50). Statistical analysis of the RT-qPCR results was performed for all samples considered separately (Student's t-test was corrected using Benjamini and Hochberg coefficient). P<0.05 was considered to indicate a statistically significant difference. The analysis was performed using the Real Statistics Resource Pack for MS Excel 2016 (Microsoft Corporation).

## Results

### 

#### Overview

Microarray analysis allows the identification of groups of genes related to the development of the nervous system and neurogenesis. Three ontological groups: ‘neurogenesis’, ‘neuronal precursor cell proliferation’ and ‘nervous system development’ were chosen. The microarray results provided 131 genes from three heat maps. The highest change in expression was demonstrated by claudin 11 (*CLDN11*), oxytocin receptor (*OXTR*), gasdermin E (*DFNA5*), ATPase phospholipid transporting 8B1 (*ATP8B1*), integrin subunit α3 (*ITGA3*), *CD9*, FRY microtubule binding protein (*FRY*), nanos C2HC-type zinc finger 1 (*NANOS1*), cysteine rich transmembrane BMP regulator 1 (*CRIM1*) and netrin 4 (*NTN4*). The first part of the results focuses on the 131 genes belonging to all three selected ontological groups. The second part of the results focuses on the genes with the highest change in expression.

#### Microarray analysis

Whole transcriptome profiling by Affymetrix microarray allowed the analysis of transcriptomic changes of the GCs during long-term *in vitro* culture after 24 h (1 day), 7, 15 and 30 days of culture. Using Affymetrix^®^ Human HgU 219 Array, the expression levels of 22,480 transcripts were examined. Genes with fold change >2 and corrected P<0.05 were considered as differentially expressed. This set of genes consisted of 2,278 different transcripts and is available as from the GEO database (https://www.ncbi.nlm.nih.gov/geo/query/acc.cgi?acc=GSE129919). The DAVID software analysis demonstrated that differentially expressed genes belong to 582 GO groups.

**Figure 1.**
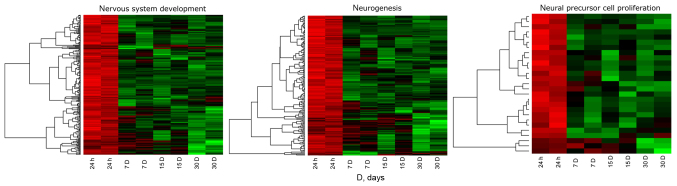
Heat map representation of differentially expressed genes belonging to the ‘neurogenesis’, ‘neuronal precursor cell proliferation’ and ‘nervous system development’ Gene Ontology biological process terms. Arbitrary signal intensity acquired from microarray analysis is represented by colors (green, higher expression; red, lower expression). Log2 signal intensity values for any single gene were resized to z-score scale (from −2, the lowest expression to +2, the highest expression for a single gene).

**Figure 2.**
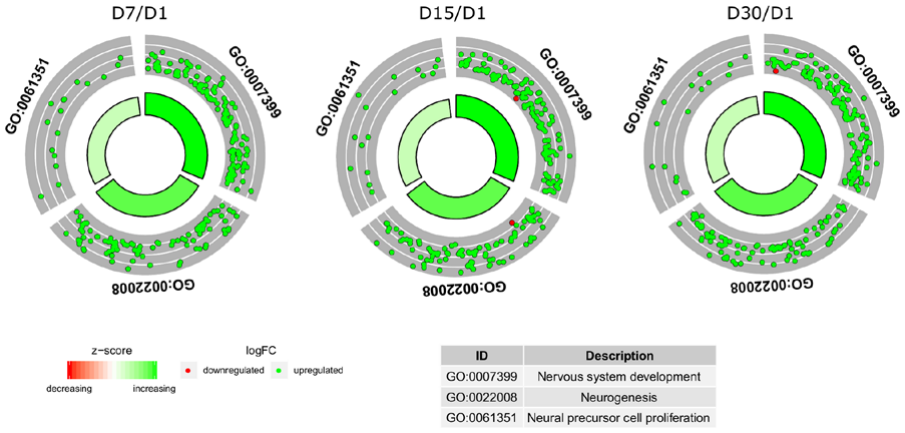
Circle plots showing the z-scores of differentially expressed genes associated with the chosen GO BP terms from the heat maps. The outer circle shows a scatter plot of the fold change in expression of the assigned genes for each term. Red circles represent downregulation and green circles represent upregulation. The inner circle shows the z-score of each GO BP term. The width of each bar corresponds to the number of genes within the GO BP term, with the color indicating the z-score. BP, biological process; FC, fold change; GO, Gene Ontology.

Fig. 1 shows the hierarchical clustering of all genes belonging to the selected ontological groups, presented as heat maps. The heat maps indicate a large number of genes involved in neurogenesis-related processes, and show the levels of expression of a given gene at particular time intervals of long-term primary *in vitro* culture. The gene symbols, fold changes in expression, Entrez gene IDs and corrected P-values of the genes are shown in Table SI. The enrichment of each GO BP term was calculated as a z-score and shown on a circle diagram (Fig. 2). The aforementioned circle graph combines data on the expression of all genes belonging to the listed ontological groups and the enrichment of gene annotations. It also demonstrates the spread of gene expression data.

**Figure 3.**
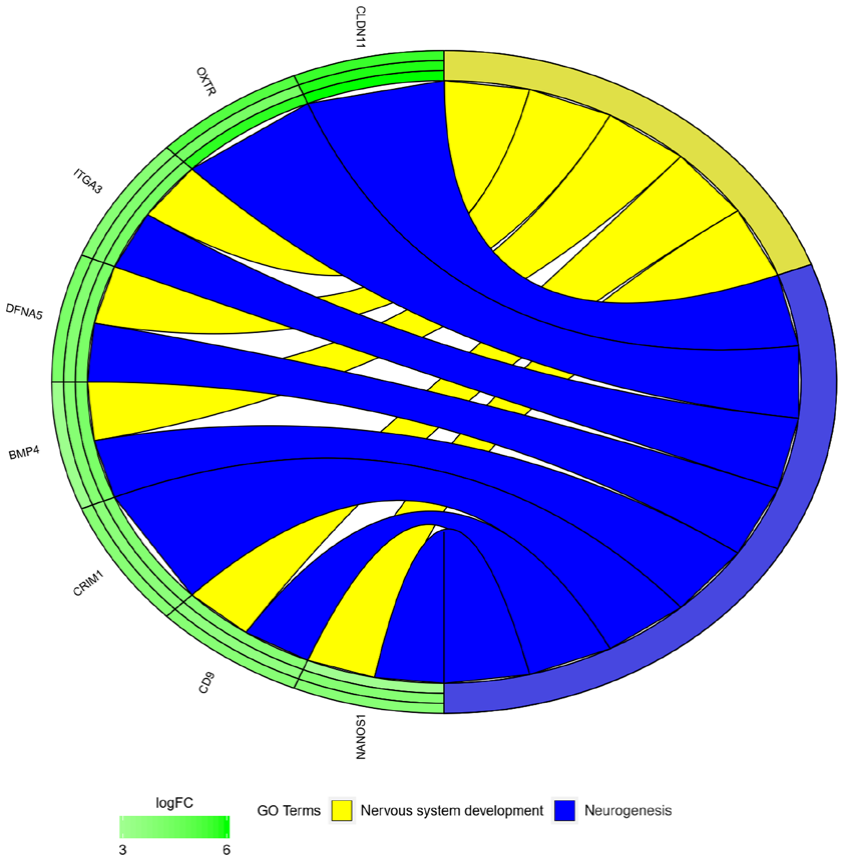
Representation of the mutual relationship between ‘neurogenesis’, and ‘nervous system development’ GO biological process terms. The ribbons indicate which gene belongs to which categories. The genes were sorted by logFC from most to least changed gene. FC, fold change; GO, Gene Ontology.

**Figure 4.**
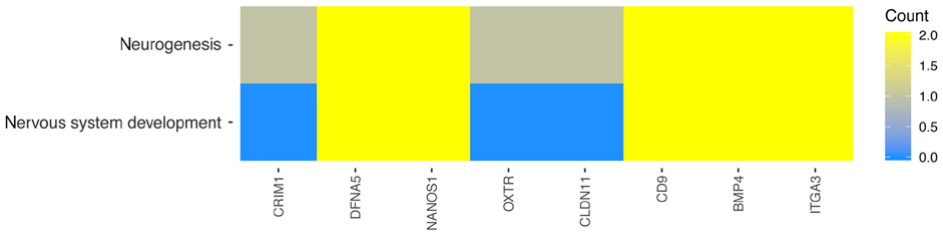
Heat map showing the gene occurrence in ‘neurogenesis’, and ‘nervous system development’ Gene Ontology process terms.

In the GO database, genes that are associated with one particular GO term can also belong to other GO term categories. For this reason, the gene intersections between the selected GO BP terms were explored. The next stage of statistical analysis was focused on analyzing the relationships between genes with the highest change in expression, closely related to the process of neurogenesis. From this group, eight genes were selected, based on their assumed importance in the process of interest. The relation between those GO BP terms was presented as a circle plot (Fig. 3) as well as heat maps (Fig. 4).

**Figure 5.**
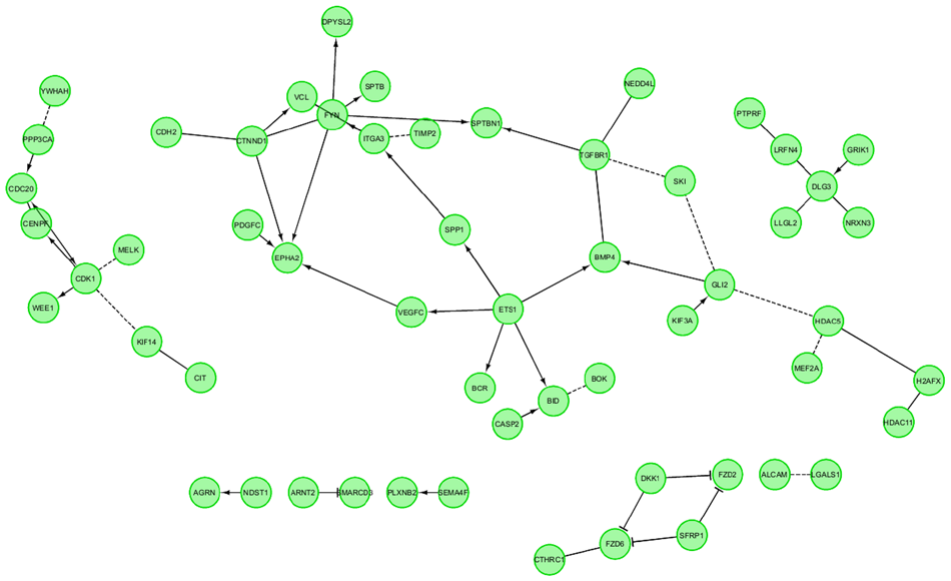
FI between chosen genes with the REACTOME FIViz application in Cytoscape 3.6.0 software. In the figure ‘->’ stands for activating/catalyzing, ‘-|’ for inhibition, ‘-’ for FIs extracted from complexes or inputs, and ‘---’ for predicted FIs. FI, functional interaction.

Finally, the FIs between chosen genes were investigated with REACTOME FIViz application in Cytoscape 3.6.0 software. This statistical analysis concerned the interaction between genes involved in neurogenesis-related processes. All genes belonging to selected ontological groups were considered in this study. The results are shown in Fig. 5. Notably, all of the genes were involved in interaction with other representatives of the analyzed group. There were four larger groups of interacting genes, as well as several genes that only exhibited singular interactions.

#### RT-qPCR results

**Figure 6.**
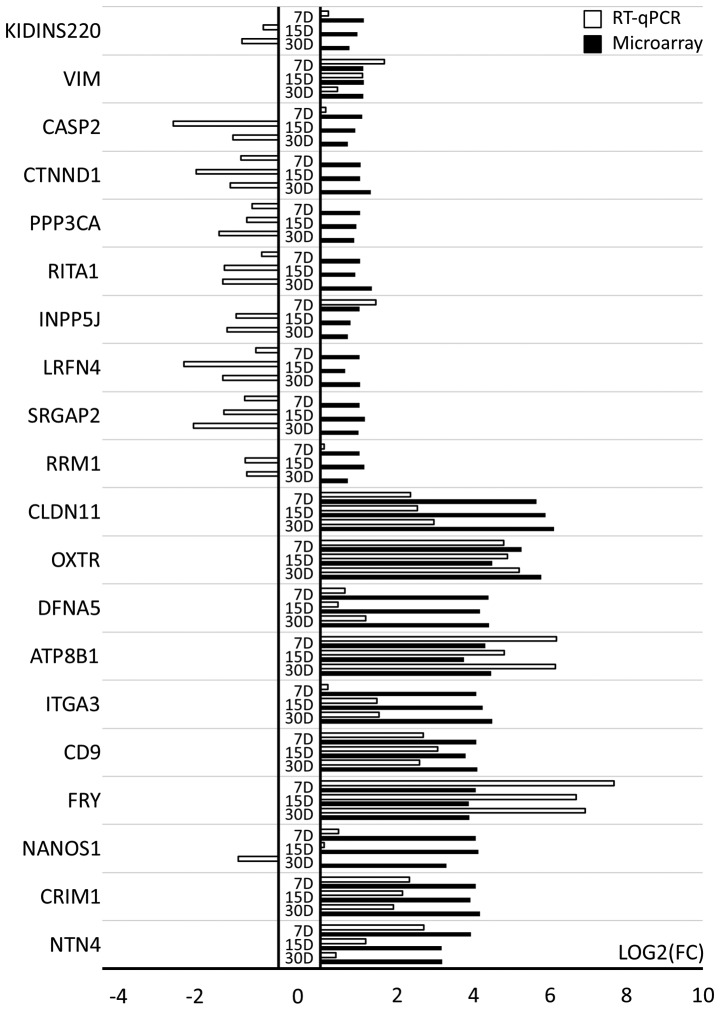
RT-qPCR results. Validation of gene expression has been presented in the form of a bar-graph. All FCs were validated in reference to the transcript levels on day 1 of *in vitro* culture. FC, fold change; RT-qPCR, reverse transcription-quantitative PCR.

The 20 genes with increased expression were subjected to validation and 10 of these genes exhibited the highest changes in expression compared to day 1 of primary culture (*CLDN11, OXTR, DFNA5, ATP8B1, ITGA3, CD9, FRY, NANOS1, CRIM1* and *NTN4*). The remaining 10 genes (*RRM1, SRGAP2, LRFN4, INPP5J, RITA1, PPP3CA, CTNND1, CASP2, VIM* and *KIDINS220*) exhibited the lowest level of upregulation, being on the border of selection for this study with a fold change only slightly >2. As shown in Fig. 6, the RT-qPCR method confirmed the direction of change in the expression of the top 10 genes with the highest transcript expression in microarray analysis in most of the cases, with the exception of *CR1M1*, which exhibited discrepancies at day 30. Differences in the scale of change are due to the sensitivity of the methods used. For some genes, the direction of change in expression was not confirmed by RT-qPCR (Fig. 6). This discrepancy can be explained in two ways: On one hand, this may be due to the fact that the RT-qPCR method is much more sensitive to quantitative changes, on the other hand it may indicate that selected genes exhibited very low upregulation of transcript levels during the microarray analysis. Such discrepancies confirm that whole transcriptomic screening requires extensive quantitative validation.

#### Cell morphology

**Figure 7.**
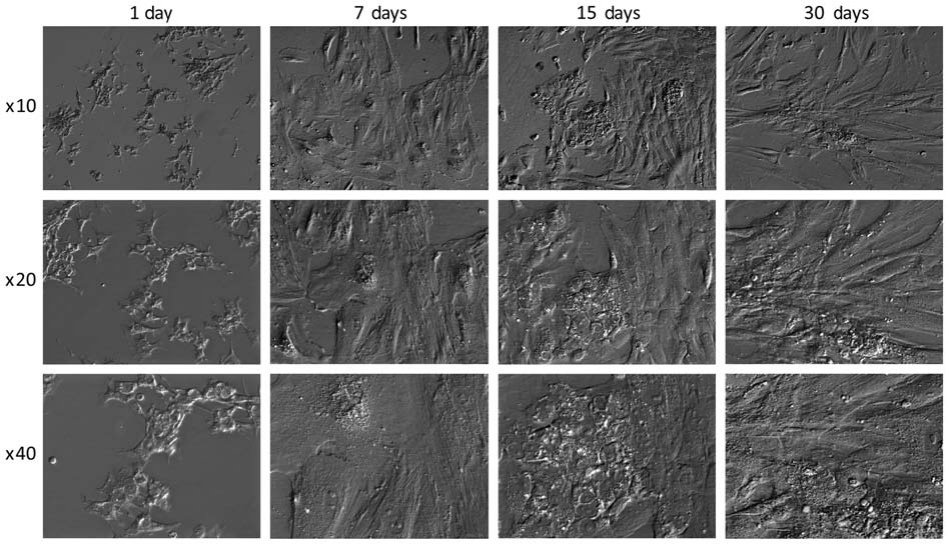
Morphology of human ovarian granulosa cells during 30 days long-term *in vitro* culture.

Notably, it has been suggested that GCs exhibit morphology similar to that of nerve cells in long-term *in vitro* culture conditions and exhibit molecular markers characteristic of neuronal cells (51). The morphology of GCs in the present study also changed. Initially, the cells had a stellate shape, after which, they transformed (regardless of confluence) into spindle-shaped cells (Fig. 7). Such changes have also been confirmed in other studies (37,39).

## Discussion

In the present study, a group of genes responsible for processes associated with neurogenesis, nervous system development and neural precursor cell proliferation were selected for analysis during long-term *in vitro* culture of GCs. The results indicated that these cells have the potential to differentiate towards neurons, as they express neural-differentiation specific genes, providing further proof for their stem-like potential (14,20). A number of studies have reported that it is possible to obtain neuronal-like cells during the differentiation/transdifferentiation of other cell types (5,39,43,52). Notably, neural-like cells can be obtained through adipocyte differentiation. These adipocyte-derived cells of neural lineage exhibited high levels of CLDN11 (oligodendrocyte-specific protein), which is a marker of glial cells (53). The *CLDN11* gene exhibited the highest expression at the individual time intervals in the present study. CLDNs are membrane proteins that belong to the peripheral myelin protein 22 superfamily. One of the most important functions of these membrane proteins is to co-create tight junction connections (54). The presence of CDLN proteins in epithelial cells and endothelium is regulated hormonally, and is also subject to changes depending on ovarian dysfunction (54). In addition, the administration of hormonal stimulation affects the expression of CLDN in the ovaries, with hCG administration causing an increase in the expression of *CLDN11* at the transcript level, which indicates the expression of this gene during ovulation (55). It is also believed that this gene may be involved in the growth of ovarian follicles in cattle (56). In addition, this gene is highly expressed in neurons (53).

Another gene that was highly expressed during the 30 days of *in vitro* culture was *OXTR*. *In vivo*, it is primarily associated with fertility and reproductive behavior (57). It has also been suggested that this gene is involved in the formation of the heart and cardiac muscle cells (58). Therefore, its expression has also been discussed in a previous study regarding the differentiation of GCs to myocardial cells (39). Many scientists believe that neurogenesis also occurs in adult mammals (59–64). Research by Lin *et al* (65) indicated that oxytocin may stimulate neurogenesis occurring in the hippocampal gyrus in adult humans. Oxytocin neurons located in the paraventricular nucleus connect directly to the hippocampus (65). Oxytocin is assumed to perform a key role in the neurogenesis of adult mammals, including humans (65). However, another study indicated that *OXTR* is not expressed in neuronal progenitor cells derived from the subgranular dentate gyrus (66).

Another gene with high expression in this study was *DFNA5*. This gene encodes proteins located in a number of organs, including the cortex of the brain and the ovaries (67–70). In the cortex, this gene is expressed at RNA and protein levels. A previous study by Croes *et al* (71) suggested that *DFNA5* may be considered a breast cancer biomarker, as there are significant differences between the expression of *DFNA5* in women with breast cancer and healthy controls. It has also been shown that the expression of this gene is influenced by the presence of the estrogen receptor (71). Other studies revealed that an increase in the expression *DFNA5* may be affected by the hormonal stimulation of patients (72–74). Assou *et al* (72) indicated that *DFNA5* expression is increased in cumulus cells obtained from patients following rFSH stimulation, compared to cells obtained following hMG-HP stimulation. These findings indicated that the high expression of *DFNA5* detected in GCs in this study may be the result of stimulation with rFSH. In the present study, patients were also stimulated with hMG-HP, so both substances may influence the aforementioned results. Previous research has indicated the possibility of the differentiation of GCs towards neurons. In addition, *DFNA5* expression is also increased in another type of MSC, in bone marrow cells during their differentiation into neuronal cells (73,74).

The *ITGA3* gene, which encodes a protein from the integrin family, was also highly expressed in GCs in this study. Integrins, as the main extracellular matrix receptors, are poorly understood in the nervous system. Non-neural tissue is the subject of the majority of research into these proteins (75). It is well known that during embryonic development, integrins serve an important role in neurogenesis (76). The extracellular matrix and its receptors, which influence the process of nerve cell formation as well as synaptic connections, seem particularly important (75). Under physiological conditions, integrins have a role in the differentiation of individual GC populations during folliculogenesis and are also present in endometrial cells (77). The results of the present study suggested that the *ITGA3* gene may be involved in the process of differentiation and formation of neuronal precursor cells. To the best of our knowledge, this study is the first to indicate this and this finding is not confirmed elsewhere in the literature.

In the present study, the *CRIM1* gene exhibited increased expression at particular time intervals of human GC culture, relative to the control (1st day of culture). Overexpression of *CRIM1* in mice increased the activity of integrins (including ITGA1), and induced phosphorylation of focal adhesion kinase and ERK. It is suggested that this gene may participate in the formation of motor neurons and regulate their viability through interaction with various growth factors. Kolle *et al* (78) reported that development of the central nervous system is dependent on the *CRIM1* gene; *CRIM1* is expressed in early motor neurons as well as in the developing spinal cord. It is believed that *CRIM1* encodes a transmembrane protein that contains the sequence of the IGF binding protein. In addition, this transmembrane protein contains numerous cysteine-rich repeats (78,79). It has also been suggested that this gene interacts with bone morphogenetic proteins (BMPs). The interaction between CRIM1 and BMP/TGFβ may be functionally important for the proper development of the central nervous system (78). In addition, it has been suggested that this gene is involved in the survival ability of motor neurons in the embryo and following injury in adult animals (80).

While not presented in detail in this study, as it was not one of the 10 genes that exhibited the biggest change in any direction, *BMP4* was also differentially expressed in GC long-term *in vitro* culture. The protein encoded by this gene serves a number of functions during embryonic and postnatal development, including bone development, mineralization, neurogenesis, adipogenesis and ovarian primordial follicle development (81–83). Co-expression of this gene with other factors (*CRIM1*) in this study suggests that long-term *in vitro* cultivation may result in the process of GC differentiation to neuronal cells. The presented results from our study (expression of *CRIM1* and *BMP4*) suggested that GCs may have the potential to differentiate into neural-like cells.

Takao *et al* (84) suggested that the expression of genes responsible for integrin formation is correlated with the presence of CD9 on surface of the GCs. In the present study regarding GCs, *CD9* and *ITGA3* were highly upregulated, but correlation studies do not show any link between them (84). CD9 is a marker of mesenchymal stem cells; a previous report demonstrated that cells expressing this marker can differentiate towards neuronal cells and express neural cell-specific proteins (vimentin, glial fibrillary acidic protein, nuclear factor, neuron specific enolase and nestin) (85). CD9 is primarily found in stem cell exosomes, and a previous study revealed that exosomes can also serve an important role in neurogenesis and can function as information relays between stem and neural cells (86). These findings suggested that the presence of this marker in GCs may indicate their potential to differentiate into neural-like cells.

The studied cells also expressed the *NANOS1* gene (87). *NANOS1* is primarily responsible for the coding of proteins involved in the development of embryonic stem cells in one or both sexes of model organisms, such as *Drosophila melanogaster* (88,89) or *Caenorhabditis elegans* (90). The human equivalent of this gene exhibits a high expression in germinal stem cells (91), as well as in oocytes at various levels of maturity (92). In addition, high expression levels of this gene have been detected in the fetal brain (93), which is important with regards to the findings of the present study.

Through analysis of the obtained results and available literature, it was concluded that GCs may possess stem-like functions and might be capable of differentiating into neuronal cells under long-term *in vitro* cultures. This suggestion was supported by the high expression of genes associated with processes, such as neurogenesis, nervous system development and neural precursor cell proliferation. The present study was carried out at the transcriptome level. Obtained results, despite validation using quantitative methods, require confirmation at the protein level, as well as an analysis of factors released during the potential differentiation process. In addition, it should be noted that analysis of the GC transcriptome using microarrays is a largely qualitative method, which can be seen in the validation carried out by RT-qPCR. Variable results might be caused by differences in microarray probe and RT-qPCR primer design, and their specificity and responsiveness to particular transcript variants. They can also result from the potential interactions between cDNAs present in the analyzed samples, leading to false positive/negative results. This further emphasizes the need of protein validation of the results, which could also account for the processes that underlie the discrepancies between transcriptomic and proteomic results, including alternative splicing, selective translation and post transcriptional regulation processes.

In conclusion, the present study analyzed the expression profile of genes belonging to three ontological groups: ‘neurogenesis’ (GO:0022008), ‘neuronal precursor cell proliferation’ (GO:0061351) and ‘nervous system development’ (GO:0007399). The results appear to support the suggestion that GCs may be able to develop into a cell line showing neuronal characteristics after 30 days of cultivation, and may serve as a basic transcriptomic entry for further research that could fully confirm this ability. Given the proven plasticity of FF GCs in the context of the current trend to consider treatment of nervous system disorders with stem cells, GCs may be considered a promising tool in the treatment of these diseases, as they appear to exhibit significant stem-like properties. Hence, it is necessary to take into account the number of mechanisms triggered by the administration of stem cells, which can be beneficial when treating diseases, due to their anti-inflammatory or immunomodulatory effects. The results of the present study are a promising introduction to further research on the mechanisms and factors that result in the ability of the analyzed cells to express neuronal features. In the future, GCs could not only become a tool of direct neuronal regeneration but also a producer of factors that may find application in the treatment of neurodegenerative diseases. Our long-term project aims to examine the further molecular mechanisms associated with genes of interest, with next stages of research planned, in which the cells in the present study will be cultured with neural differentiation medium. Furthermore, the authors will aim to compare the transcriptome of GCs before and after exposure to neural differentiation medium.

## Supplementary Material

Supporting Data

## Figures and Tables

**Table I.** Oligonucleotide sequences of primers used for reverse transcription-quantitative polymerase chain reaction analysis.

Gene	Primer sequences (5′-3′)	Product size (bp)
*NTN4*	F: GGCCTGGAAGATGATGTTGT	234
	R: TTGAGGCTCTTCGTTCAGGT	
*CRIM1*	F: GGAAGGAGAAACGTGGAACA	247
	R: GTCAGGCTTCCAGGACTCAG	
*NANOS1*	F: GCTCCTGGAACGACTACCTG	209
	R: GTCGTCGTCCTCGTCGTAGT	
*FRY*	F: CCAGCACAGTGACCTCTCAA	232
	R: AACAAGGACGTTGGAGTTGG	
*CD9*	F: TTGGTGATATTCGCCATTGA	160
	R: ACGCATAGTGGATGGCTTTC	
*ITGA3*	F: GCCTGCCAAGCTAATGAGAC	247
	R: AGAAGCTTTGTAGCCGGTGA	
*ATP8B1*	F: TGCATACGAGGATTGGTTCA	189
	R: ACCCCATGCAACAAGCTTAC	
*DFNA5*	F: AGGTGGCTTCGAGAACAAGA	234
	R: AATAGGACCGCCTGGAAGAT	
*OXTR*	F: TTCTTCGTGCAGATGTGGAG	234
	R: GGACGAGTTGCTCTTTTTGC	
*CLDN11*	F: CTGGTGGACATCCTCATCCT	190
	R: CCAGCAGAATGAGCAAAACA	
*RPM1*	F: GGAGGAATTGGTGTTGCTGT	235
	R: GCTGCTCTTCCTTTCCTGTG	
*SRGAP2*	F: ACTAAAGGAGGCGGAGAAGC	220
	R: GTACTCATTCCGGGCTTTGA	
*LRFN4*	F: GGACTGGTGGACCTGACACT	194
	R: GATGAGGTGCTGCAGATTGA	
*INPP5J*	F: TTCAACTTCGTGCTGGTGAG	248
	R: TTCAGGAAGCAGAGCATGTG	
*RITA1*	F: CCCTCACACCAAGGAAGAAG	204
	R: CTCTGTCTTGGAGGGACCAG	
*PPP3CA*	F: TGCATCAATTCTTCGACAGG	162
	R: AAGGCCCACAAATACAGCAC	
*CTNND1*	F: TCTGCCATAGCTGACCTCCT	208
	R: GGAGTTCTGCTGTCCTCCTG	
*CASP2*	F: GACGCAGGATATTGGGAGTG	170
	R: GGCAGCAAGTTGAGGAGTTC	
*VIM*	F: GAGAACTTTGCCGTTGAAGC	199
	R: TCCAGCAGCTTCCTGTAGGT	
*KIDINS220*	F: CTGATGATAGCTGCCGAACA	191
	R: GAGCTGTCCATCCTCCCATA	
*RRM1*	F: GGAGGAATTGGTGTTGCTGT	235
	R: GCTGCTCTTCCTTTCCTGTG	
*GAPDH*	F:TCAGCCGCATCTTCTTTTGC	90
	R:ACGACCAAATCCGTTGACTC	
β-actin	F:AAAGACCTGTACGCCAACAC	132
	R:CTCAGGAGGAGCAATGATCTTG	
*HPRT1*	F:TGGCGTCGTGATTAGTGATG	141
	R:ACATCTCGAGCAAGACGTTC	

F, forward; R, reverse.

## Data Availability

The datasets used and/or analyzed during the current study are available from the corresponding author on reasonable request. In addition, the datasets generated and/or analyzed during the current study are available in the Gene Expression Omnibus repository, [https://www.ncbi.nlm.nih.gov/geo/query/acc.cgi?acc=GSE129919].
