# Supplementary material for: Expression of genes involved in neurogenesis, and neuronal precursor cell proliferation and development: Novel pathways of human ovarian granulosa cell differentiation and transdifferentiation capability in vitro
Source: Mol Med Rep. 2020 Jan 31;21(4):1749–60. doi: 10.3892/mmr.2020.10972 (PMC7057781; doi:10.3892/mmr.2020.10972)
Supplement: Supporting Data [file Supplementary_Data.pdf]

Table SI. Gene symbols, fold changes in expression, Entrez gene IDs and corrected P-values of studied genes.

Gene symbol	Fold change D1/D7	Fold change D1/D15	Fold change D1/D30	Adjusted P-value D1/D7	Adjusted P-value D1/D15	Adjusted P-value D1/D30	Entrezgene ID
RRM1	2.021768	2.206091	1.636671	0.101894	0.047511	0.029857	6240
SRGAP2	2.024766	2.246311	1.986332	0.397802	0.031081	0.018244	23380
LRFN4	2.032537	1.56422	2.050337	0.093162	0.031081	0.102524	78999
INPP5J	2.037824	1.725832	1.634614	0.701629	0.030205	0.05892	27124
RITA1	2.045079	1.877727	2.524093	0.07383	0.03106	0.040661	84934
PPP3CA	2.05061	1.913019	1.851225	0.801999	0.023258	0.028641	5530
CTNND1	2.07606	2.059376	2.493493	0.053947	0.005581	0.005289	1500
CASP2	2.130807	1.871144	1.640512	0.341741	0.021112	0.033354	835
VIM	2.16462	2.195607	2.168564	0.898534	0.007447	0.006371	7431
KIDINS220	2.184925	1.958272	1.685647	0.218728	0.011981	0.016759	57498
TGFBR1	2.188996	1.585418	1.46801	0.607609	0.026412	0.106546	7046
LGALS1	2.194273	2.541993	2.831199	0.367503	0.012251	0.006371	3956
KIF3A	2.19478	1.850005	2.377716	0.120395	0.024245	0.04543	11127
NCS1	2.2783	2.131948	3.105525	0.083209	0.047675	0.053779	23413
UST	2.286128	2.010455	3.614628	0.011059	0.033646	0.049568	10090
MAFK	2.295558	1.799692	1.958553	0.636237	0.037734	0.090026	7975
CDH2	2.31119	1.986439	1.92385	0.790919	0.01156	0.018732	1000
ARHGEF10	2.311249	2.788637	2.663974	0.762668	0.022675	0.010843	9639
APBB2	2.369929	2.427177	1.940409	0.066329	0.006592	0.00555	323
VEGFC	2.371705	2.424384	2.313969	0.645536	0.005848	0.004928	7424
LLGL2	2.377292	1.636103	1.254952	0.167014	0.032817	0.138612	3993
OXCT1	2.382383	1.80451	1.980136	0.339645	0.004574	0.013521	5019
GRIK1	2.383064	1.193713	0.890863	0.143759	0.035814	0.622602	2897
DLG3	2.436979	2.551924	2.641258	0.828416	0.021919	0.016638	1741
SKI	2.46271	2.50275	3.341721	0.009359	0.002709	0.002478	6497
SPTB	2.495918	1.027184	1.131062	0.309574	0.003611	0.900817	6710
BCR	2.507611	2.251619	2.333804	0.796931	0.013329	0.017344	613
NDST1	2.511612	1.661929	2.158918	0.238363	0.045663	0.189784	3340
PDGFC	2.511784	2.418348	1.507838	0.036645	0.031252	0.031879	56034
SMARCD3	2.528987	2.267934	1.75636	0.085402	0.010645	0.013543	6604
ALDH5A1	2.558353	2.314292	1.677168	0.05644	0.013554	0.016975	7915
ALCAM	2.60493	2.795375	2.424953	0.370225	0.015947	0.011234	214
CIT	2.640185	2.962862	2.672979	0.651374	0.049375	0.031522	11113
DDR1	2.662986	2.94323	3.572125	0.220422	0.013283	0.008572	780
RAPGEF2	2.699117	2.710037	2.220224	0.229365	0.014947	0.012877	9693
PTPRF	2.702878	4.770853	4.265291	0.577868	0.03106	0.006932	5792
CHODL	2.702979	1.178002	2.376011	0.001895	0.010781	0.544138	140578
HDAC11	2.7084	2.836511	3.386951	0.440859	0.044834	0.034765	79885
ETV5	2.708423	2.852748	3.23393	0.579209	0.044049	0.033713	2119
KIAA1524	2.717902	2.241218	2.636196	0.388991	0.023517	0.040249	57650
SRD5A1	2.760316	2.128315	2.577624	0.355952	0.029719	0.063092	6715
PREX1	2.76119	2.010922	2.47796	0.225131	0.015567	0.042697	57580
FYN	2.765514	2.90417	2.632699	0.611206	0.025738	0.020094	2534
AMIGO2	2.822957	2.242601	1.994635	0.590445	0.034722	0.064087	347902
H2AFX	2.909718	2.789465	2.46423	0.59303	0.038499	0.038925	3014
PLXNB2	2.946326	3.461029	3.859933	0.530261	0.015373	0.008749	23654
MACF1	2.96498	2.495056	3.184634	0.179	0.014122	0.021586	23499
MAP4	3.013993	2.897716	3.017057	0.550918	0.000975	0.001052	4134
MEF2A	3.019241	3.647594	2.798678	0.05228	0.00411	0.002502	4205
INPP5F	3.042892	2.209785	3.136749	0.08512	0.015904	0.039283	22876
MYRF	3.065858	2.714928	2.634595	0.739561	0.002201	0.002579	745
BID	3.13175	2.87727	4.106849	0.097974	0.018015	0.020233	637
NR2F2	3.151739	4.366718	9.598608	0.002299	0.011103	0.004543	7026
MYO5A	3.192165	2.99197	2.953521	0.921307	0.00532	0.005812	4644
CAPRIN1	3.265348	2.703495	3.057969	0.607973	0.031458	0.047338	4076
EHD1	3.288062	2.32814	2.36694	0.925579	0.012729	0.032111	10938
PMP22	3.295479	2.73516	2.78587	0.913533	0.011073	0.016304	5376

Table SI. Continued.

Gene symbol	Fold change D1/D7	Fold change D1/D15	Fold change D1/D30	Adjusted P-value D1/D7	Adjusted P-value D1/D15	Adjusted P-value D1/D30	Entrezgene ID
SPTBN1	3.304848	2.030568	2.916961	0.077568	0.012773	0.054511	6711
NEGR1	3.305925	2.757526	7.004404	6.76E-05	0.002435	0.003428	257194
AGRN	3.309808	3.519888	3.736432	0.713208	0.009464	0.007131	375790
CTHRC1	3.347823	4.023322	6.926104	0.07344	0.039801	0.023368	115908
FKBP1B	3.387376	3.237422	3.318001	0.78001	0.001599	0.001564	2281
SPINT2	3.398409	2.431264	1.779229	0.296779	0.049554	0.106068	10653
DAB2IP	3.416795	3.13563	2.853393	0.58502	0.010395	0.011322	153090
MAP1B	3.423203	2.404338	3.267402	0.150889	0.01537	0.038091	4131
NEDD4L	3.476711	4.917672	3.080767	0.026445	0.009286	0.003902	23327
CADM1	3.538659	3.216645	2.079601	0.105235	0.026393	0.03	23705
MELK	3.610806	3.068771	2.326444	0.279117	0.026413	0.035583	9833
CDK6	3.72549	3.761877	4.232504	0.630469	0.024327	0.021586	1021
FZD2	3.767679	3.278544	13.84389	6.05E-05	0.005716	0.007268	2535
SEMA4F	3.786062	4.162757	3.646759	0.583079	0.022269	0.015942	10505
HDAC5	3.786869	2.483511	2.146236	0.370811	0.006147	0.017928	10014
ATL1	4.035	2.978216	3.397891	0.152485	0.000966	0.001702	51062
WLS	4.245624	3.487205	5.789078	0.052296	0.01351	0.01881	79971
ENC1	4.245979	4.134794	3.264972	0.309849	0.014021	0.013059	8507
DBN1	4.712013	3.825961	5.302759	0.085375	0.004498	0.006368	1627
RACGAP1	4.735836	5.297663	5.276126	0.988633	0.024108	0.017461	29127
MAOB	4.747149	3.859033	2.809173	0.097754	0.004713	0.006671	4129
NDRG4	4.817497	7.098036	3.324764	0.055552	0.037583	0.017119	65009
EPHA2	4.8202	2.854057	3.518869	0.174434	0.002778	0.008316	1969
VCL	4.864573	3.380753	5.783599	0.089052	0.020792	0.04079	7414
YWHAH	4.894869	4.460129	4.853179	0.554234	0.002681	0.002934	7533
SFRP1	5.256953	6.418839	5.765165	0.630468	0.009045	0.005699	6422
TUBB3	5.308206	3.291997	4.796059	0.156564	0.011903	0.029655	10381
SULF2	5.382245	8.025055	2.158912	0.005145	0.026926	0.012415	55959
FZD6	5.4593	6.975979	6.816073	0.930615	0.015024	0.008617	8323
TACC3	5.531522	6.683994	5.408581	0.378566	0.009647	0.006215	10460
STIL	5.6288	5.40042	4.900424	0.716835	0.014125	0.01345	6491
GRIP1	5.726394	4.406289	4.064269	0.718133	0.007818	0.011576	23426
KIF14	5.876355	6.330966	5.326401	0.526469	0.01338	0.010384	9928
ETS1	5.91011	7.886957	6.458214	0.473602	0.01398	0.007791	2113
DHFR	6.100375	6.873142	4.669302	0.005748	0.000681	0.000531	1719
TIMP2	6.200145	7.209695	8.681742	0.06879	0.00054	0.000453	7077
BOK	6.252747	10.38335	9.720958	0.825161	0.016775	0.006967	666
APLP1	6.724176	11.70933	10.39692	0.567198	0.004562	0.002177	333
DCLK1	6.825138	7.638479	5.965188	0.304473	0.006409	0.004872	9201
GLI2	6.93552	7.103782	14.11276	0.072541	0.019199	0.016304	2736
PRICKLE1	7.301909	4.726273	9.353689	0.042893	0.010666	0.020201	144165
NRXN3	7.989118	14.67486	31.63559	0.107869	0.033342	0.013494	9369
DPYSL2	8.828498	15.39167	20.07619	0.157396	0.0021	0.001052	1808
DKK1	8.954961	9.635247	10.15533	0.777067	0.002435	0.002043	22943
FAM150B	8.963014	7.693274	15.96865	0.073842	0.016194	0.017889	285016
CENPF	9.293946	12.37396	10.64819	0.545598	0.004852	0.003253	1063
SPP1	9.788134	0.95252	2.839958	0.040754	0.027852	0.965318	6696
DIXDC1	10.19146	9.163015	15.31045	0.011713	0.001194	0.001266	85458
BMP4	10.48954	17.22155	21.24347	0.611556	0.020467	0.010072	652
HMGB2	10.66394	11.73624	10.31678	0.516114	0.002329	0.001834	3148
SSTR1	11.71047	17.24349	25.57376	0.213809	0.006481	0.003779	6751
ANKRD1	11.88737	2.508097	2.617912	0.925684	0.024792	0.27876	27063
NDRG1	11.92954	22.62052	20.31863	0.594171	0.002201	0.001102	10397
WEE1	12.58023	12.68202	11.20957	0.693251	0.00688	0.006215	7465
SH3GL3	12.80461	5.936084	8.941457	0.106988	0.002681	0.006872	6457
TNFRSF21	13.29178	11.52699	19.84632	0.305804	0.029183	0.03114	27242
ANXA3	13.40458	19.42721	21.68463	0.821712	0.025705	0.015167	306

Table SI. Continued.

Gene symbol	Fold change D1/D7	Fold change D1/D15	Fold change D1/D30	Adjusted P-value D1/D7	Adjusted P-value D1/D15	Adjusted P-value D1/D30	Entrezgene ID
TWIST1	13.41066	16.06133	20.74144	0.178018	0.001349	0.001064	7291
CDK1	13.88075	18.56825	12.19633	0.085503	0.002233	0.001491	983
GAS6	13.92343	14.85424	26.70902	0.093827	0.005943	0.005065	2621
STMN1	14.15491	16.77585	16.17827	0.887561	0.003396	0.002666	3925
ARNT2	14.51122	12.33988	16.76564	0.353657	0.006258	0.006839	9915
CDC20	14.63169	16.17955	15.95021	0.970839	0.011951	0.009375	991
ASPM	15.06876	21.27444	15.88402	0.511297	0.016229	0.009871	259266
NTN4	15.2823	9.026144	9.0834	0.976884	0.002192	0.003428	59277
CRIM1	16.63216	15.16397	17.96461	0.422677	0.001635	0.00157	51232
NANOS1	16.71367	17.47953	9.848209	0.047777	0.002489	0.002264	340719
FRY	16.7709	14.68067	14.90368	0.971378	0.012828	0.013034	10129
CD9	16.80523	13.90259	17.17638	0.306295	0.00152	0.00157	928
ITGA3	16.92781	18.84218	22.47009	0.74729	0.027709	0.022255	3675
ATP8B1	19.91928	13.4672	22.05294	0.127331	0.003449	0.00471	5205
DFNA5	21.0067	17.97532	21.3502	0.429055	0.001486	0.001498	1687
OXTR	38.38617	22.60881	54.85452	0.002981	0.00068	0.001012	5021
CLDN11	50.24748	59.01065	68.98347	0.799101	0.014803	0.011576	5010
